# Reconstruction of the Odontoid Process by a Tricortical Iliac Crest Graft in a Case of Tuberculosis of C1, C2, and C3 Vertebrae

**DOI:** 10.7759/cureus.742

**Published:** 2016-08-19

**Authors:** Paresh Golwala, Chirag Kapoor, Aditya Merh, Maulik Jhaveri

**Affiliations:** 1 Orthopaedics, Sumandeep Vidyapeeth, Vadodara, Gujarat

**Keywords:** cervical tuberculosis, iliac crest graft, odontoid process, cervical plate

## Abstract

Tuberculosis (TB) is an emerging disease which affects about one-third of the world’s population, especially in developing countries. TB of the spine is the most common type of skeletal TB. Cervical spine TB is rare, constituting 2-3% of all cases of spinal TB. We would like to present an unusual case of tuberculosis of the C1, C2, and C3 vertebrae with neurological deficit and its difficult management. A new method of treatment was done for this patient, which included reconstruction of the odontoid process using a tricortical iliac crest graft that was fixed with an anterior cervical plate. On follow-up, there was good incorporation of the graft. The neurological condition of the patient improved and was normal with partial restriction of neck movements. We suggest this technique to be worthwhile for treatment of this disease at this location.

## Introduction

Tuberculosis (TB) is an emerging disease that affects about one-third of the world’s population, especially in developing countries [1]. Skeletal TB constitutes 3-5% of all cases of TB, with TB of the spine being the most common [2]. Cervical spine TB is rare, constituting 2-3% of cases of spinal TB, but consequences are serious as death can occur due to cord compression secondary to atlantoaxial dislocation [3]. Upper cervical TB accounts for only about 1% of all cases of spinal TB [4]. It carries a significant risk of neurological involvement [5], and hence, diagnosis and intervention are necessary.

Lesions of the odontoid process in Pott’s disease affecting the cervical spine are very unusual and range from minor damage to bone destruction, leading to spinal instability.

The different lines of management include anti-tuberculous drugs with traction and halo-pelvic fixation or adjunctive surgery (debridement and stabilization) in patients with severe or persistent neurological complications/vertebral instability.

We would like to present an unusual case of tuberculosis of C1, C2, and C3 vertebrae with neurological deficit and its management. To our knowledge, this is the first such reported case in the literature.

## Case presentation

A 17-year-old female patient presented with pain and restricted movements in the neck for the previous three months. The pain was insidious at onset, dull in nature, and was increasing gradually. She also had a low-grade fever, which was relieved with medication. She was treated conservatively elsewhere but had only temporary relief and movements were still restricted. She was diagnosed with pulmonary tuberculosis 15 days prior to presentation for which AKT Category I had been started. She presented to us with a four-day history of weakness and tingling sensation in the left upper limb and torticollis. The patient agreed to participate and was explained the nature and objectives of this study, and informed consent was formally obtained. No reference to the patient's identity was made at any stage during data analysis or in the report.

On examination, tenderness was present in the posterior aspect of the upper neck region. The neck was in spasm with torticollis and chin deviated towards left side. Motor power was Grade III in the left shoulder, elbow, and wrist joint. Finger grip was weak with hypoesthesia in the left palmar region.

**Figure 1:**
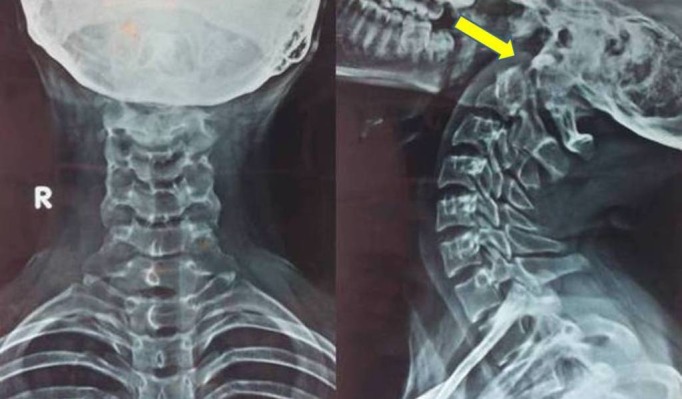
X-Ray Anteroposterior View (left) and Lateral View (right) of the Cervical Spine Subluxation of the C1 vertebra over the C2 vertebra (arrow).

An X-ray revealed subluxation between the C1 and C2 vertebrae, but no lesion was seen (Figure 1).

**Figure 2:**
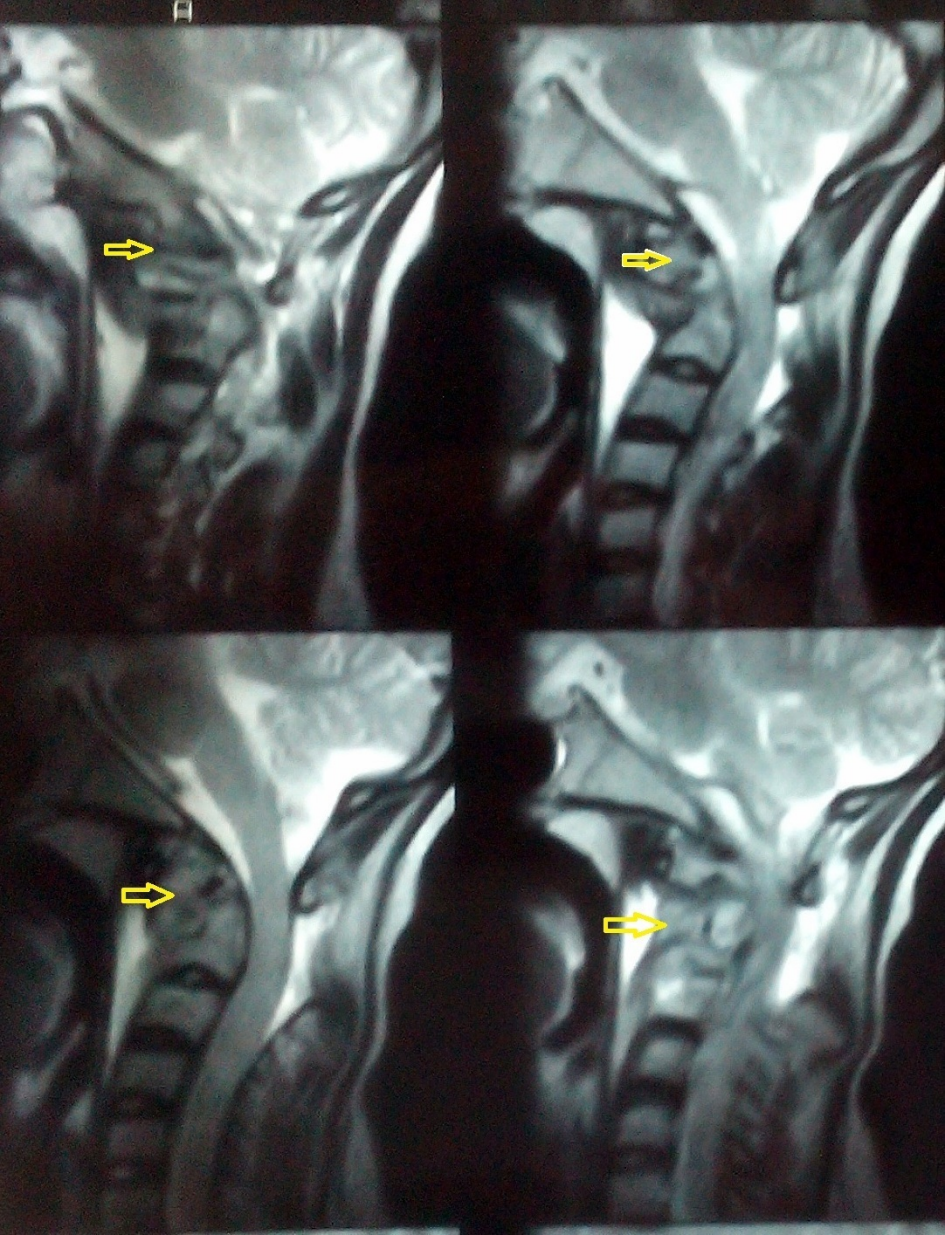
MRI of the Cervical Spine T2 weighted images showing lesions in the C1, C2, and C3 vertebrae (arrows) with resorption of the odontoid process.

On MRI, there were lesions in the C1, C2, and C3 vertebrae with resorption of the odontoid process of the C2 vertebra, and anterior translation of C1 over C2 vertebral body causing compression of the upper cervical cord. Cord compression at the C2-C3 and C3-C4 levels was present with paravertebral abscess, suggesting Koch's disease (Figure 2).

**Figure 3:**
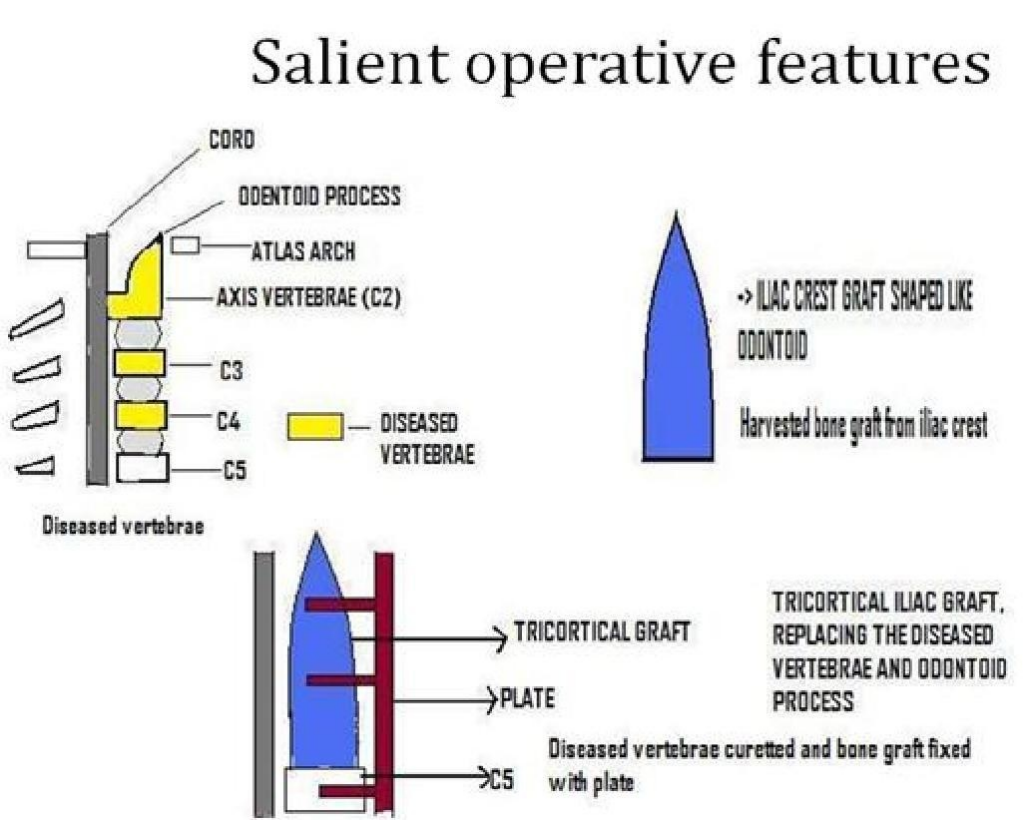
Schematic Representation of the Procedure Performed An iliac crest graft was shaped like the odontoid process, put in its place, and fixed with a cervical plate.

**Figure 4:**
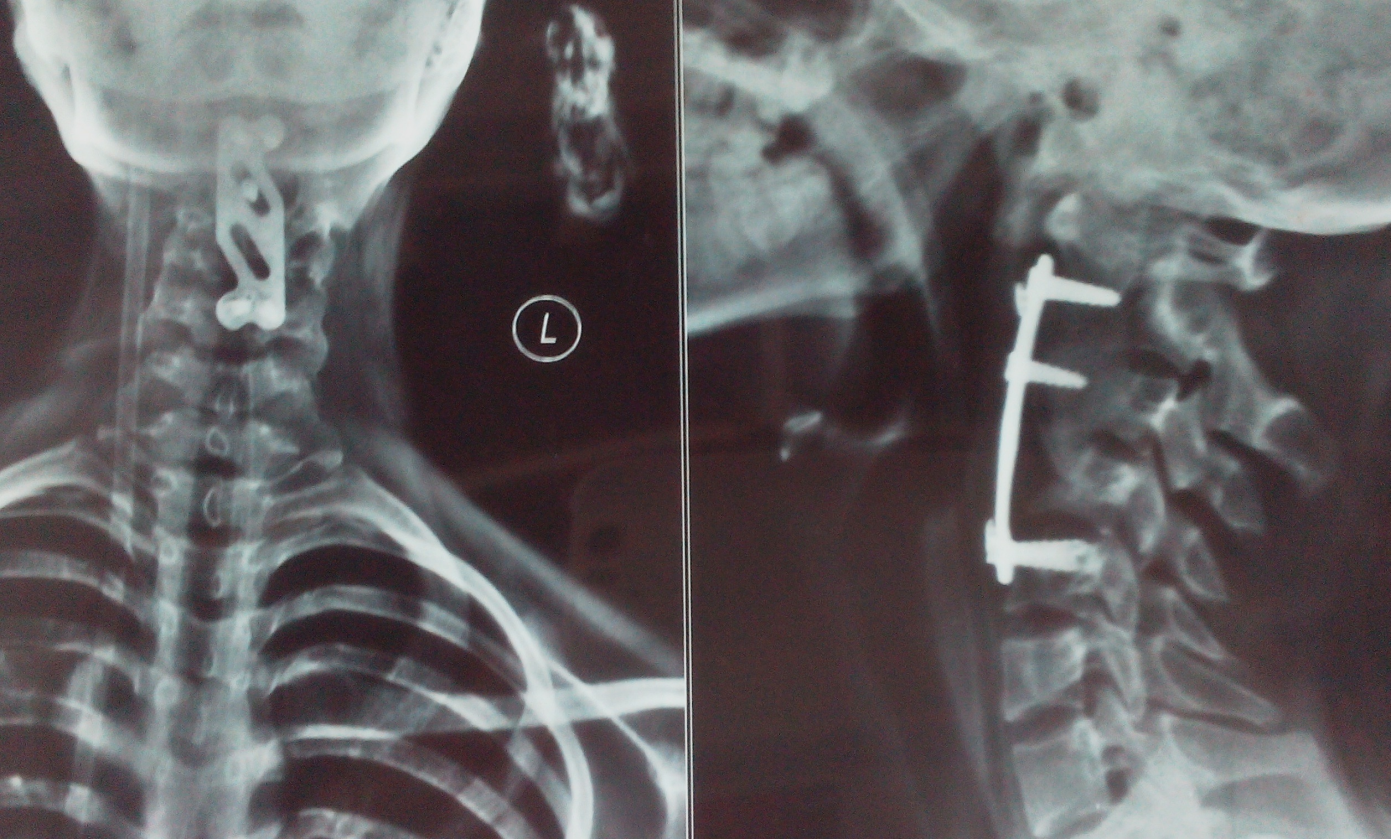
Postoperative X-rays, Anteroposterior View (left) and Lateral View (right) of the Cervical Spine Reconstruction of the odontoid process by a tricortical iliac crest graft and fixed with anterior cervical plate.

The patient was on AKT since the last one month. Streptomycin was added, and after explaining all risks for the treatment, the patient was taken to surgery. A retropharyngeal approach from the anterolateral aspect of the neck was taken, and the cervical vertebrae were exposed. The cold abscess was posterior to the esophagus and anterior to the odontoid process. The odontoid process was severely eroded with extension into C1 and C2 bodies. The upper half of the C3 vertebra was also eroded. After curetting the diseased tissue, a void was left extending right from the arch of the atlas to almost the entire body of C3. The length of the void was obtained, which was found to be about 6 cm. The tricortical graft obtained from the iliac crest was shaped as the odontoid process and was fixed in the void created and stabilized by an anterior cervical locking plate. Two screws were passed in the graft and two in the normal vertebral body. The posterior surface of the arch of the atlas was denuded so that fusion could occur between the graft and the C1 vertebral body (Figures 3-4).

A wide dissection was necessary in front of the vertebrae, which was achieved by displacing the esophagus. The patient had dysphagia postoperatively, and a Ryle’s tube was placed for a period of six weeks following surgery.

Immobilization was done with a Philadelphia collar, but it led to a subluxation of C1-C2 seen at her two-month postoperative visit because the patient failed to comply with its proper use.

**Figure 5:**
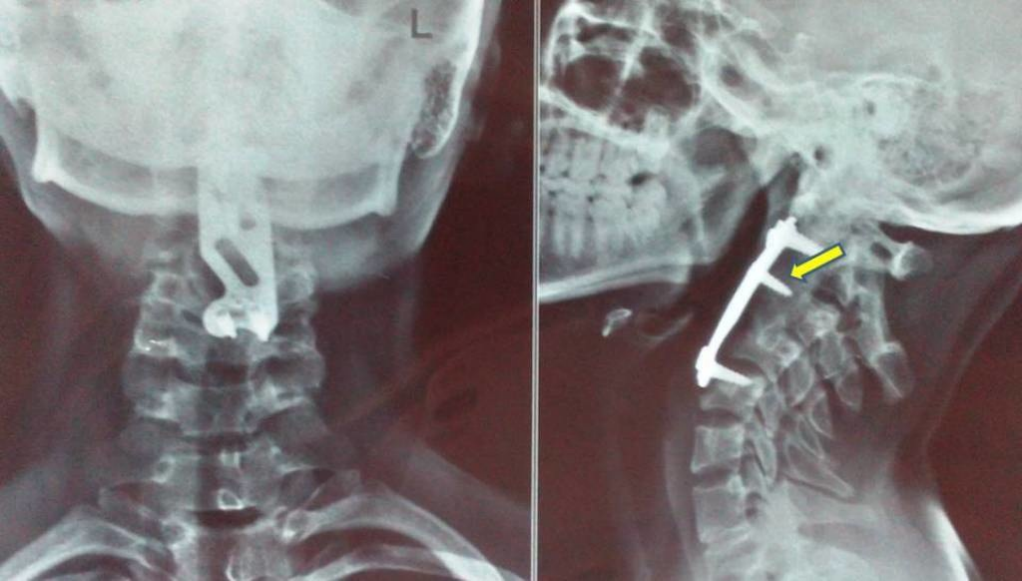
Follow-Up X-Rays, Anteroposterior View (left) and Lateral View (right) of the Cervical Spine Good incorporation of the tricortical iliac crest graft (arrow).

On final follow-up after 18 months, radiographs showed that the graft was incorporated with a mild subluxation of C1 over the C2 vertebrae (Figure 5).

Clinically, the patient had restricted neck movements with no residual neurological deficit, i.e., Frankel Grade E (full motor and sensory recovery), and had no difficulty in swallowing.

## Discussion

Tuberculosis of the upper cervical spine is seen during the first three decades of life and seen equally in both sexes. The clinical picture ranges from nonspecific symptoms to severe neurological complications and death because of instability and cord compression. As the disease progresses, it starts involving the ligamentous structures nearby and also causes osteolytic erosion of the odontoid process or the C1 vertebra, which may lead to subluxation. The spinal cord at the upper cervical region can be damaged by vertebral subluxation, compression by an abscess, or by direct invasion of tuberculous bacilli so it should be treated urgently [6].

The rarity of this condition is responsible for the lack of clear guidelines for its management. Both the conservative and operative approaches for such patients have been proposed in the literature, each having merits and demerits. To avoid late onset neurological compression, it is better to prevent deformity. Conservative management includes giving traction to the patient for a prolonged duration, but it has disadvantages like keeping the patient bedridden and its sequelae, which are cumbersome to the patient. Persistent instability can cause pain and progressive neurological deficit. Patients who are treated conservatively have an average of 15-degree kyphosis, and 3% to 5% of these patients have a deformity of greater than 60 degrees, especially seen in young patients in whom bone growth leads to worsening of the deformity [7]. If surgery must be done in a conservatively treated patient at a later date after the lesion has healed, it is technically very difficult.

The lesions involving the vertebrae and pedicles of the upper cervical spine cause instability [8] and require anterior instrumentation to stabilize the spine, which has been shown to be associated with a high fusion rate, low complication rate, and increased correction rate of kyphosis. In 1975, Tuli proposed a "middle-path" regimen for the treatment of spinal TB consisting of conservative treatment with multidrug chemotherapy and surgery for specific indications [9]. We followed that regimen for this patient as there was neurological deterioration and an increasing risk for subluxation of C1 and C2 vertebrae, making reconstruction of the odontoid process necessary. Since the vertebral body lies anteriorly and is commonly affected by tuberculosis, decompression and stabilization should be done by approaching the spine anteriorly [10]. Posterior instrumentation and stabilization is another option in these patients; however, it does not deal with the disease process at all.

As the literature does not have any specific guidelines, a newer method was adopted in this patient to fill the void created after removing the diseased tissue by a tricortical iliac crest graft resembling the excised tissue. The tricortical iliac crest graft has following advantages:

1. It gives the strength required due to its tricortical structure.

2. It gets incorporated faster due to its cancellous structure.

3. It can be shaped according to our requirements and easily harvested for the required length.

4. It is held well with the cervical spine plate and, thus, helps in stabilization of the column.

The benefit of spinal instrumentation is immediate postoperative stability before fusion occurs. Anterior cervical plating is associated with a number of complications, which include dysphagia, injury to the recurrent laryngeal nerve, cord injury, and vascular injury during exposure. Dysphagia after anterior cervical surgery is as high as 60% [11], and our patient also had dysphagia for six weeks probably because of extensive dissection and collateral damage.

Neurological recovery following anterior surgical decompression and fusion is not always satisfactory as some amount of residual kyphosis remains; however, in our case, the patient had a complete neurological recovery by three months. Loembe, et al. have reported a reduction of kyphosis in only four patients in a series of nine adult patients with cervical tuberculosis with cord compression treated by anterior debridement and fusion [12]. However, there have been recent reports in the literature about the satisfactory results obtained in anterior fixation of the cervical spine in tuberculosis using a plate and screws with excellent correction of the kyphosis and strong stability [13]. This was also seen in our patient, and there was no kyphosis present postoperatively. Nevertheless, due to improper immobilization, there was a mild translation of C1 over C2.

The rate of graft failure is quite high, i.e., 59%. The graft may also sink into the cancellous vertebral bodies causing exaggeration of kyphosis, especially in patients with involvement of two or more vertebrae. This was not seen in our patient, and radiologically, the graft was seen to be incorporated on follow-up.

## Conclusions

In addition to decompression, anterior reconstruction with a tricortical iliac crest graft and fixed with locking cervical plate is a useful method for the prevention and correction of the deformity in the lesions involving the odontoid process. The tricortical iliac crest graft provides a good, viable option for reconstruction of odontoid. A satisfactory segmental stability and fusion are achieved.

## References

[1] Murray CJ, Styblo K, Rouillon A (1990). Tuberculosis in developing countries: burden, intervention and cost. Bull Int Union Tuberc Lung Dis.

[2] Fang D, Leong JC, Fang HS (1983). Tuberculosis of the upper cervical spine. J Bone Joint Surg Br.

[3] Lukhele M (1996). Tuberculosis of the cervical spine. S Afr Med J.

[4] Kanaan IU, Ellis M, Safi T, Al Kawi MZ, Coates R (1999). Craniocervical junction tuberculosis: a rare but dangerous disease. Surg Neurol.

[5] Dhammi IK, Singh S, Jain AK (2001). Hemiplegic/monoplegic presentation of cervical spine (C1-C2) tuberculosis. Eur Spine J.

[6] Fang D, Leong JC, Fang HS (1983). Tuberculosis of the upper cervical spine. J Bone Joint Surg Br.

[7] Rajasekaran S (2002). The problem of deformity in spinal tuberculosis. Clin Orthop Relat Res.

[8] Tuli SM (1974). Tuberculosis of the craniovertebral region. Clin Orthop Relat Res.

[9] Tuli SM (1975). Results of treatment of spinal tuberculosis by “middle-path” regime. J Bone Joint Surg Br.

[10] Jain AK (2002). Treatment of tuberculosis of the spine with neurological complications. Clin Orthop Relat Res.

[11] Govender S (2002). The outcome of allografts and anterior instrumentation in spinal tuberculosis. Clin Orthop Relat Res.

[12] Loembe PM, Mwanyombet-Ompounga L, Assengone-Zeh Y, Kengue-Lechiombeka PR (2000). Early anterolateral surgery for tuberculosis of the lower cervical spine with neurological complications in adult. Our experience in Gabon (Article in French). Neurochirurgie.

[13] Li L, Cui SQ, Wang H (2004). Clinical application of anterior cervical locking plate systems and analysis of complications as well as their countermeasures. Zhongguo Xiu Fu Chong Jian Wai Ke Za Zhi.

